# Filicide in Bangladesh: A case indicating the need for psychosocial support among mothers during peripartum

**DOI:** 10.1002/jgf2.624

**Published:** 2023-04-18

**Authors:** S. M. Yasir Arafat, Shirajum Monira, Shakila Ashfia Lily

**Affiliations:** ^1^ Department of Psychiatry Enam Medical College and Hospital Dhaka Bangladesh; ^2^ Department of Pharmacology Enam Medical College Dhaka Bangladesh; ^3^ Department of Forensic Medicine and Toxicology Enam Medical College Dhaka Bangladesh

**Keywords:** filicide, personality disorder, psychosocial support, suicide prevention

## Abstract

We report a case of filicide which is an under‐researched entity in Bangladesh. A 28‐year‐old lady visited with complaints of irregular eating followed by self‐induced vomiting, poor anger control, irregular sleep, hopelessness, and suicidal thoughts for the last year. On the third visit, she admitted that she killed her 32‐day‐old baby by keeping it in a refrigerator. The case raises some forensic psychiatric complexities as the patient confessed it to the psychiatrist while family members know it as an accidental aspiration. It indicates the complex nature and dire need for psychosocial support in Bangladesh.

## Abstract 2

We report a case of filicide which is an under‐researched entity in Bangladesh. The case raises some forensic psychiatric complexities as the patient confessed it to the psychiatrist while family members know it as an accidental aspiration. It indicates the complex nature and dire need for psychosocial support during pregnancy and peripartum in Bangladesh.

## 1 INTRODUCTION

Filicide, the perpetration of killing a child by its parents is a highly complex entity in forensic psychiatry. The association of different psychosocial factors is being revealed eventually behind this deliberate act which compels mental health professionals and caregivers to be aware of overcoming the emerging challenges both on the medico‐legal ground and for therapeutic purposes.^1^ Various mental illnesses have been reported among the indicted parents including delusional disorder, borderline personality disorder, affective disorder (with or without psychosis), and substance disuse.^2^, ^3^ Battering, throttling, drowning, stabbing (with the sharp cutting pointed weapon), and suffocation are the commonly used methods to execute filicide.^1^ This deliberate killing becomes more miserable when the perpetrator is the mother rather than the father.^4^

As a result of the complex nature and overlapping perspectives, extensive studies are warranted to identify the risk factors for filicide. However, it is an under‐researched entity in Bangladesh. The psychosocial aspects considering the prevention of filicide have been poorly addressed in South Asian countries, especially Bangladesh. Here, we present a case of a 28‐year‐old woman suffering from anorexia nervosa, borderline personality disorder, and chronic major depressive disorder who has regretfully confessed to killing her 32‐day‐old daughter. The case depicts a complex forensic psychiatric dilemma and the need for psychosocial support during pregnancy in Bangladesh.

## 2 CASE PRESENTATION

A 28‐year‐old well‐educated, beautiful lady hailing from a city along with her husband visited a psychiatrist at a tertiary care hospital. Her complaints were irregular eating followed by self‐induced vomiting, poor anger control, irregular sleep, hopelessness, and suicidal thoughts for the last year. The couple had a daughter who died on her 32nd day of life 1 year ago and currently, the couple was not interested to carry out further pregnancy. She had depressive symptoms for the last 5 years that continued during her pregnancy. Three months after the death of the child, she availed psychiatric consultations and psychotherapy sessions while she was non‐compliant. Her symptoms were not reduced during the consultation. She had a traumatic childhood with a bad parental relationship and a forceful marriage by her parents from which she fled. She has a history of multiple non‐fatal suicide attempts by multiple methods. She married 5 years ago following a love affair; however, currently, most of the time she lives alone as her husband remains busy with his job and she has no active job or social life. Based on a detailed history and mental state examination, she was diagnosed with a case of anorexia nervosa, borderline personality disorder, and dysthymia. She has been treated with fluoxetine 20 mg daily in the morning, lamotrigine 75 mg with gradual up‐titration, and doxepin 6 mg with sleep hygiene and breath‐holding relaxation exercise. She was suggested inter‐personal psychotherapy along with medications.

She started improving with the medications and attending psychotherapy. On the third visit, while she was discussing her unwillingness to further pregnancy she admitted that she killed her baby which gives her enduring trauma. She thought that she would not tolerate the pain again which may indulge her to kill her baby again. She experienced a few bouts of crying while she was describing the event. No other physical and/or emotional changes were noticed during the session and she returned to her home after it. She killed the baby by keeping it in the refrigerator for about 3 min while she was alone at home with her baby. After 3 min regretting of the sudden incident, she took her baby to the hospital but it was already dead. She has a bad relationship with her in‐laws as they do not approve their affair marriage. The child was unexpected and the couple tried to abort the fetus but could not help. She regarded that child as a curse. Also, there was no supportive caregiver when the baby was born. She was depressed and frustrated with her changed physique because of the pregnancy while she wanted to visit a psychiatrist which was disallowed by her husband and in‐laws.

## 3 DISCUSSION

The case indicates the complex nature of filicide and the need for psychosocial support during pregnancy and after delivery in Bangladesh. The patient was suffering from several psychiatric disorders which were not addressed during her antenatal check‐up indicating negligence toward psychiatric services. For the enduring stigma toward mental illness, the treatment of psychiatric disorders is often neglected by patients, family members, and even gynecologists during pregnancy in Bangladesh. Additionally, the country lacks a well‐effective referral system for psychiatric care. There is also a high out‐of‐pocket expense. In the next phase, the patient was deprived of family support after the delivery.

Our case was a lady with a high risk of self‐destructive behavior because of her unfavorable circumstances like traumatic childhood, bad parenting, forceful marriage to another person, psychiatric morbidities, bad relationship with in‐laws, and inadequate support from her husband. Additionally, despite her request, they did not attend a psychiatrist or avail mental health support at that time. With a newborn unexpected child, having changes in body physique, no psychosocial help, and own bad childhood (insecure attachment) may force her to the event. The method of this filicide is unusual, that is, keeping the baby in the refrigerator. We identified a previous extraordinary case of filicide in Bangladesh perpetrated by a university student while she delivered her baby in the university residential hall and she hide the baby in a trunk to avoid disclosure in 2019.^5^ A previous report identified that a filicidal mother is either suffering from mental unsoundness or affected severely by hormonal ups and downs at least for a brief period of time.^6^ Different psychiatric disorders have been observed among those mothers.^3^ Like many other cases, this case reveals that the patient had an unwanted child also she was diagnosed as a case of borderline personality disorder, chronic depressive disorder, and anorexia nervosa.^1^, ^7^ The patient had a history of non‐fatal attempts multiple times which is also supported by previous reports as filicide has been regarded as a part of extended suicide.^2^, ^8^

We speculate several areas for the prevention of such filicide in Bangladesh. First, enquiring about mental and referring to mental health care during the antenatal check‐up would reduce the suffering. A good awareness among obstetricians and gynecologists is warranted to maintain effective collaboration with psychiatrists. Second, care from the immediate family members reduces the distress and identifies the risky suicidal, homicidal, and filicidal thoughts which can prevent such attempts. Third, the implementation of social policies should be considered to stop child abuse in Bangladesh. Marriage after an affair is an important cultural conflict in Bangladesh. It is time for the social reappraisal of it and reduces emotional conflicts within the family.

At the same time, the case raises some forensic psychiatric complexities as the patient confessed it to the psychiatrist while her family members know it as an accidental aspiration. Professional dilemmas arise about whether to disclose the issue to the family members and her spouse or to report the case to the police while the patient requests confidentiality. Upon considering the social context of Bangladesh, her mental unsoundness, rational pre‐existing history, and current treatment status were not disclosed to the legal authority by the physician. A similar dilemma was also encountered by a psychiatrist previously in Bangladesh while managing persons with active suicidal intents.^9^ Further observation is warranted regarding these complex situations. However, we speculate that forensic psychiatrists could consider possible alternatives considering the cultural norms. A similar challenge has also been indicated that filicide has been a challenge both in psychiatric evaluation and in taking legal actions.^10^ Along with long‐term psychotherapy and psychopharmacological support, good social support, helpful family bonding, proper supervision, and checking for vulnerability factors might be helpful to prevent filicide and extended suicides.^3^

## 4 CONCLUSION

Our case indicates the complex nature of filicide and a dire need for collaborative support in Bangladesh. There is an urgent need for adequate psychosocial support for mental health problems during pregnancy and peripartum periods for a healthy mother and a healthy child. Enduring collaboration is warranted among psychiatrists, forensic medicine, gynecologist, pediatricians, and general physicians along with other stakeholders.

## AUTHOR CONTRIBUTIONS

SMYA: Conception, data collection, writing, and reviewing. SM: writing. SAL: writing.

## CONFLICT OF INTEREST STATEMENT

The authors declare that they have no conflict of interest to report.

## ETHICS APPROVAL STATEMENT

This case has been reported in accordance with the Helsinki Declaration of 1975.

## PATIENT CONSENT STATEMENT

Informed written consent was taken before submitting the manuscript.

## Data Availability

Data sharing is not applicable to this article as no datasets were generated or analyzed during the current study.
